# Influence of Shoe Characteristics on the Development of Valgus Foot in Children

**DOI:** 10.3390/jcm8010085

**Published:** 2019-01-12

**Authors:** Miguel Medina-Alcantara, Jose Miguel Morales-Asencio, Ana María Jimenez-Cebrian, Joaquin Paez-Moguer, Jose Antonio Cervera-Marin, Gabriel Gijon-Nogueron, Ana Belen Ortega-Avila

**Affiliations:** 1 Department Nursing and Podiatry, University of Malaga, 29071 Malaga, Spain; migmedalc@uma.es (M.M.-A.); amjimenezc@uma.es (A.M.J.-C.); joaquinpaez@uma.es (J.P.-M.); jacervera@uma.es (J.A.C.-M.); anaortavi@uma.es (A.B.O.-A.); 2 Instituto de Investigación Biomédica de Málaga (IBIMA), 29010 Malaga, Spain

**Keywords:** child, flatfoot, shoe, school, foot disease

## Abstract

For thousands of years, shoes have been worn to protect the feet from injury, and the proper choice and use of footwear are directly relevant to foot health, especially that of children. The aim of this study was to evaluate the association between shoe-related factors (type and frequency of use) and the prevalence of valgus foot in children. This analytical cross-sectional observational study was carried out on a population of children in the first, second or third year of primary education, to analyzing the frequency and type of shoes worn, and to determining the presence or not of valgus foot. The sample consisted of 132 children (of 642 potential subjects), with an average age of 7.53 years (Standard Deviation (SD) 0.80), which was composed of 61 boys (46.2%) and 71 girls (53.8%). The overall prevalence of valgus foot was 45.5% (*n* = 60). The use of boots 2–5 days a week was significantly associated, in both sexes, with a lower prevalence of valgus in the left foot (30.5%, *p* = 0.009). The use of boots could be associated with a lower presence of valgus, depending on the frequency of wear.

## 1. Introduction

The child’s foot is constantly growing and solidifying its structure and form. The morphological and functional development of the foot is influenced by internal (gender, genetics) and also external (e.g., shoewearing habits) factors. During growth, childrens’ feet react more sensitively, especially to external factors, and are therefore subjected to greater traumatic stresses than adult feet. The importance of accurate fitting is essential in children, since their foot structure is not consolidated and the influence of compression might be harmful [1]. 

Several studies have validated that ill-fitting shoes can impede the normal development of the maturing foot and cause foot problems and pathologies in childhood and adulthood [2,3].

For children, the correct choice of footwear is especially important because their growth and development are characterised by the evolutionary dynamics of the locomotor system and by the physical activity to which the lower body is subjected. However, this choice is often influenced by aesthetic, economic, or marketing issues, rather than by considerations of health. To raise awareness of the importance of these questions, and to enable parents and others to reach suitable decisions, research is needed to highlight the direct influence of the shoe on the movement of the foot and on its functionality [3]. 

The design of children’s shoes should be based on the barefoot model, prioritising impact absorption and load distribution, in the understanding that overly rigid and/or tight-fitting footwear can provoke injuries or deformities [2,3,4].

To date, no universally accepted definition of paediatric valgus foot (PVF), or flatfoot, has been established. This condition consists of a valgus deviation of the hindfoot associated with a decrease or flattening of the internal longitudinal arch. PVF varies considerably, and it can be painful or non-painful, flexible or rigid, functional or non-functional [5,6]. Pathological or rigid PVF is often characterised by stiffness of the foot, which provokes disability and requires treatment, while physiological or flexible PVF is a normal variation that does not cause any disability and tends to improve over time [6,7]. 

Estimates of the prevalence of PVF vary widely, from 0.6% to 77.9%, reflecting the absence of generally-accepted criteria with which to differentiate a pathological foot from a normal one, and the lack of consensus on defining this condition. The estimated prevalence among pre-school children is about 45%, which decreases to 15% among older children (by the age of 10 years). In other words, PVF normally becomes less acute as the child grows [8,9]. 

Many factors have been associated with the risk of PVF, including excess body weight, age, sex, race, joint laxity, place of residence, and physical activity, but the choice and use of footwear is known to be of major significance in this respect [8,9,10,11,12,13]. 

Many studies have examined how footwear may provoke deformities and an increased incidence of PVF. Rigid footwear restricts the mobility of the metatarso-phalangeal joints and it may contribute to the development of a pathological foot. Flexible shoes, barefoot walking on uneven terrain, and exercises that enhance the plantar muscles all stimulate proprioceptive sensitivity, favour neuromuscular development, and reduce the incidence of PVF [14]. Walking barefoot during childhood is also believed to strengthen the extrinsic and intrinsic musculature of the foot, providing dynamic support for the long arch [7]. 

Rao et al. [15] observed that the prevalence of PVF was correlated with the use of shoes. Thus, it was present in 8.6% of the children who wore shoes, but in only 2.8% of those who did not. This study was carried out in the Indian city of Mandalore, on a sample of 2300 children that were aged 4–13 years, of whom habitually 1555 wore shoes, while 745 never did so, due to their low socioeconomic status.

Sachithanandam et al. [16] studied the possible influence of footwear on PVF and reported an incidence of 3.24% among those who started wearing shoes before the age of six years, of 3.27% in those who started between the ages of 6 and 15 years and of 1.75% in those who first wore shoes at the age of 16 years or later.

Tong et al. [17] examined the relationship between the use of different types of footwear and the development of the internal longitudinal arch (ILA). In a cohort study of 111 healthy children with an average age of 6.9 years, these authors used pressure platforms to measure the arch index (the average contact area of the foot, divided by the total area, excluding the toes), the average peak pressure exerted by the foot and the maximum force, normalised by body weight. Follow-up examinations that were conducted after 10 and 22 months led the authors to conclude that the type of footwear used during childhood can influence the development of ILA and that children who wore closed shoes had a flatter ILA than those whose feet were more exposed.

However, less is known about the effects of the frequency of use and the type of shoes worn by children. Accordingly, the aim of the present study is to determine the association between the shoe factor (type and frequency of use) and the prevalence of PVF among children.

## 2. Methods

An observational cross-sectional analytical study was conducted on a population of children in the first, second, and third years of primary education, during the school year 2012–2013, at five schools in the city of Malaga (Spain). The following inclusion criteria were applied: (i) aged 6–9 years, (ii) informed consent provided by parent or guardian. Parents/guardians were previously informed about the study and completed a questionnaire with the data required on the participants. The exclusion criteria were history of a surgical procedure being performed on the foot or the existence of a congenital malformation.

The sample size was determined by application of the EPIDAT program to detect a prevalence of PVF of 44% (20), for an eligible population of *n* = 700 children, with an alpha value of 0.05, and a precision of 8%, was *n* = 123 subjects. 

**Figure 1:**
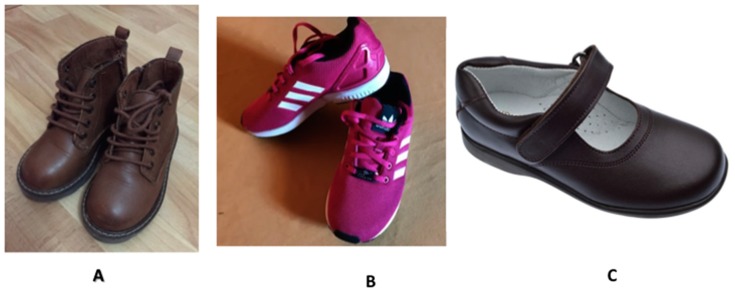
Classification of type of shoes used in the study: (**A**) Boot (**B**) Sport shoes (**C**) Street shoes.

The main study variables were the presence of valgus, the child’s age (in months, obtained from the school’s administrative record) and sex, together with detailed information on the type of shoe worn and the frequency of use (Figure 1).

For the diagnosis of PVF, although it is frequently associated with flatfoot, we evaluated the valgus deviation of the hindfoot by reference to the valgus index [18], which is calculated from an in situ pedigraphy obtained of each child’s feet, differentiating the left foot from the right. The pedigraphy was taken while using an ink pedigraph, with the child in a standing position, arms beside the body and both feet resting on a flat surface. The base angle was adjusted to fit the dimensions of the pedigraph. 

**Figure 2:**
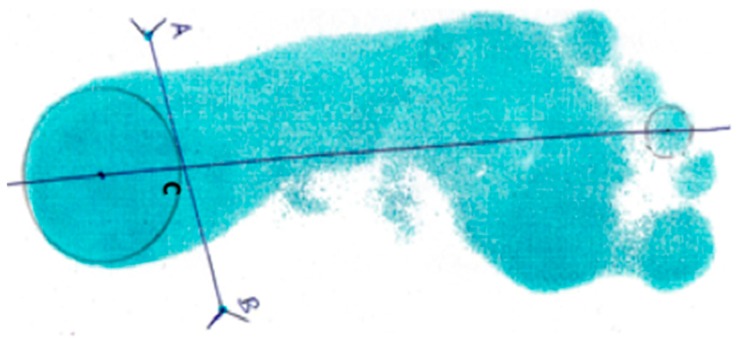
Measure of the Valgus Index with the following formula (1/2AB − AC) × 100/AB.

The valgus index was analysed on the pedigraph by reference to the study by Thomson [18]. The positions of each malleolus were marked on the plantar impression, where Point A represented the external malleolus and Point B the internal one, and the two points were connected by a straight line. The axis of the foot was then drawn, with a straight line from the centre of the heel to the mark made by the middle toe. The intersection between the line A–B and the foot axis is called point C. From these points, the following formula can be applied to determine the valgus index: (1/2AB − AC) × 100/AB (Figure 2). The valgus index was assessed by a podiatrist (MMA) with a high previously established intra-rater reliability for Valgus Index scoring (intraclass correlation coefficient (ICC) = 0.91–0.98), who was blinded to the purposes of the study and to the participant’s identity. 

The values obtained were classified into three types of posture: flatfoot/pronation (>14°); physiological flatfoot and normal (11–14°); and, supinated foot (<11°).

The type of footwear was classed as sports shoes, street shoes or boots, and the frequency of use was divided into three categories: 1 day/week, 2–5 days/week, >5 days/week). These data were obtained from questionnaires that were addressed to the children’s parents or guardians. 

The characteristics of footwear would depend not only on its type, but also on the relationship to the wearer (or foot) and the expected function. Mc Poil et al. [19] identified seven generic shoes can be classified: oxford, pump, clog, mule, moccasin (street shoes), defined like a flexible to allow for foot mobility, and bend under the ball of the foot. Offers resistance to twisting, boot like a worn to provide additional ankle support and have a heel that is distinguishable from the rest of the sole and sport shoes, it has a flexible sole, appropriate tread for the function, and the ability to absorb impact [20].

### 2.1. Analysis

To preserve the independence of data [21], and based on the strong correlation between Foot Posture Index scores for left and right feet achieved in previous studies [22], although both were measured, for further statistical analysis only one foot (the left, chosen at random) was included in the statistical analyses.

The descriptive statistics obtained were measures of central tendency (mean, median) and dispersion (standard deviation and interquartile range) for the quantitative variables, depending on the normality of the distribution of the variables, which was verified by the Kolmogorov-Smirnov test and by estimating the asymmetry and kurtosis of the distributions. Association analysis was performed using the chi-square test, with Fisher’s adjustment if necessary, and by calculating the odds ratio and the corresponding 95% confidence intervals. All of the analyses were carried out using SPSS V.22 software (IBM SPSS Statistics for Windows, Version 22.0; IBM Corp, Armonk, New York, NY, USA).

### 2.2. Ethical Issues

This study was authorised by the Ethical & Research Committee of Malaga (Spain) (25.02.2016). Informed consent was asked to parents or legal tutors. The study complies with the principles that were laid down in the Declaration of Helsinki.

## 3. Results

The sample consisted of 132 children (of 642 potential subjects), whose average age was 7.53 years (SD (Standard Deviation) 0.80). Of these children, 61 (46.2%) were boys and 71 (53.8%) were girls. By age group, 32.7% were in the first year of primary school, 25% in the second year, and 41.3% in the third year.

The overall prevalence of PVF (flatfoot/pronation > 14°) by valgus index was 45.5% (*n* = 60). As regards symmetry, the valgus was more accentuated in the right foot, with a prevalence of 41.7%, in contrast to 39.4% in the left. Differences were also found by sex, with pathological valgus being measured more frequently among the boys, both in the left foot (odds ratio (OR) for girls: 0.35; 95% confidence interval (CI): 0.17 to 0.73) and in the right (OR for girls: 0.43: 95% CI: 0.21 to 0.88) (Table 1).

Our analysis of the relationship between valgus foot and the type of footwear used revealed a significant difference only for the boys and girls who wore boots 2–5 days a week, who had less presence of valgus both in the left foot, and in the average valgus index. The other study factors that were considered did not present statistical significance (Table 2).

## 4. Discussion

Our hypothesis is that there may be a relation between the shoe factor (type and frequency of use) and the prevalence of PVF among children. If this is so, further study should be undertaken to design a multidisciplinary health action protocol and thus guide the selection of the type of footwear in children. This study addresses an issue that is of great interest in a wide range of fields, especially in podiatry, but which has only recently attracted research attention.

Our analysis of the relationship between valgus foot and the type of footwear used revealed a significant difference only for the boys and girls who wore boots 2–5 days a week, who had less presence of valgus both in the left foot, and were in the average valgus index (57.89%).

This issue could be explained because, among the boys aged 6 to 7 years, the feet were more pronated than the girls of the same age. This is accounted for by the fact that, at this age, the foot is still developing and evolves naturally from a flattened (pronated) posture to a more neutral one. Since girls develop earlier than boys, they seem to have already completed this stage of development and thus present lower valgus foot scores than the boys [23,24].

Previous studies have identified various factors that influence the appearance of PVF, such as the type of shoe worn, excess body weight, age, sex, race, joint laxity, place of residence, and physical activity [8,9,10,11,12,13,25]. The effects of footwear on foot development have focused primarily on the morphology of the medial longitudinal arch [15,26], but this has suggested that the mechanisms to cope with perturbations to standard gait occur later once gait is less variable and is more sensitive to factors, such as footwear. All reported work has been undertaken in cultures where shod walking is commonplace and therefore the influence on habitually barefoot infants and children is unquantified [26,27,28].

The prevalence of 45.5% recorded in our study is within the wide range of estimates made in this respect, from 0.6% [29] to 77.9% [30] and is close to the 44% reported by Pfeiffer et al. [31] in 2005. However, our results are much higher than the 2.7% observed by García-Rodríguez et al. in 1999 and the 28% of Chen et al. [12] in 2009, but well below the 58.7% of Chen [32] and the 59% of Chang et al. [33]. This heterogeneity highlights the need to standardise evaluation methods and to conduct multicentre studies to enable international comparisons to be made, with similar criteria. Future research efforts, therefore, should focus on developing consensus recommendations on the measurement of the paediatric foot, using valid and reliable instruments for this purpose [34]. Concretely, important aspects of the child’s foot, such as the concept of what a normal foot, is in relation to age and body development and consequently the modification of foot posture [21].

A child’s gait becomes established at around five years of age [35], and it is influenced by various factors, including (in developed economies) the type of shoes worn [36]. However, in determining the association between valgus foot and the type of footwear used, most studies only distinguish between the use or otherwise of footwear [14,15]. To our knowledge, only one previous study has differentiated the type of footwear and its relationship with the development of the foot [17]. These authors found that children who wore closed shoes had a flatter ILA than those who wore more open shoes. In our study, children who wore boots 2–5 days a week had a lower prevalence of valgus in the left foot, which is possibly due to the medial area of the shoe containing or reducing forces that would otherwise deform the ALI, thus reinforcing the structure of the foot. Moreover, this type of shoes could contribute to fix the rotation across the subtalar joint axis. However, Staheli suggest that stiff and compressive footwear may cause deformity, weakness, and loss of mobility [2].

Nevertheless, these results that were obtained in the left foot might be influenced by the dominance of the lower limb which has shown differences in plantar pressures between right and left foot in healthy school children [37]. On the other hand, boot usage is very influenced by environmental factors, and these results should be tested in other settings with different patterns of wearing boots. In countries with warm weather, such as Spain, boots are only used in winter season, therefore, studies in countries with other climate conditions should be developed. Nonetheless, the average valgus index showed a significant statistical difference in children who wore boots more frequently. Likewise, the results show each foot valgus index and global index and it would be interesting to compare with different type of shoes.

A possible limitation of the present study arises from its cross-sectional design. Although different consecutive ages were included in the study population, we were unable to detect relationships with a significant strength of association. Longitudinal studies are needed to further our understanding of these questions. Moreover, the study approach that is described here could usefully be extended to include other types of footwear and intensities of use. 

The clinical implication of this study is to help direct in the selection of appropriate footwear for children. For example, our study shows that the use of boots may be a preventative factor against the development of PVF, however we should consider these results in relation to the limitations previously mentioned, and it could be beneficial to do a clinical trial to reaffirm the hypothesis 

## 5. Conclusions

The use of boots 2–5 days a week was significantly associated, in both sexes, with a lower prevalence of valgus and could be associated with a lower presence of valgus, depending on the intensity of use.

## Figures and Tables

**Table 1:** Characteristics of the sample. SD: Standard Deviation.

	Male (*n* = 61; 46.2%)	Female (*n* = 71; 53.8%)	
	*n* (%) or mean (SD)	*n* (%) or mean (SD)	*p*
Age	7.53 (0.75)	7.54 (0.85)	0.996
Valgus index (Right)	32 (52.50)	23 (32.4)	0.022
Valgus index (Left)	32 (52.50)	20 (20.80)	0.007
Valgus index (Average)	33 (54.1)	27 (38.0)	0.047

**Table 2:** Type and frequency of footwear and valgus index.

	Sports Shoes					Street Shoes		Boots	
	Valgus Index (Left) *n* (%)								
	No	Yes	Total	No	Yes	Total	No	Yes	Total
1 day/week	5 (6.6)	3 (6.1)	8 (6.4)	16 (24.6)	14 (32.6)	30 (27.8)	16 (39)	13 (72.2)	16 (39)
2–5 days/week	52 (68.4)	33 (67.3)	85 (68)	44 (67.7)	24 (55.8)	68 (63)	24 (58.5)	3 (16.7)	24 (58.5)
>5 days/week	19 (25.0)	13 (26.5)	32 (25.6)	5 (7.7)	5 (11.6)	10 (9.3)	1 (2.4)	2 (11.1)	1 (2.4)
Total	76 (60.8)	49 (39.2)	125	65 (60.2)	43 (39.80)	108	41 (69.5)	18 (30.5)	59
	*p* = 0.979			*p* = 0.449			*p* = 0.009		
